# Characterization of temperature-sensitive *Schizosaccharomyces pombe* mutants in the septation initiation network Spg1 GTPase

**DOI:** 10.17912/micropub.biology.001193

**Published:** 2024-06-12

**Authors:** Anna Bowman Fletcher, Lesley A. Turner, Liping Ren, Alaina H. Willet, Kathleen L. Gould

**Affiliations:** 1 Department of Cell and Developmental Biology, Vanderbilt University School of Medicine, Nashville, TN, US; 2 The Harpeth Hall School, Nashville, TN, US

## Abstract

The *Schizosaccharomyces pombe* GTPase, Spg1 , activates the septation initiation network (SIN) protein kinase cascade to trigger septation. In the absence of functional Spg1 , cells fail cytokinesis and become multinucleate. In this study, we characterize a set of temperature-sensitive *spg1* alleles isolated in the 1990s. We identify the mutations within each new and previously characterized allele, characterize the extent of relative growth defects, and assess their interaction with other SIN alleles.

**Figure 1. Characterization of spg1 mutant alleles:**
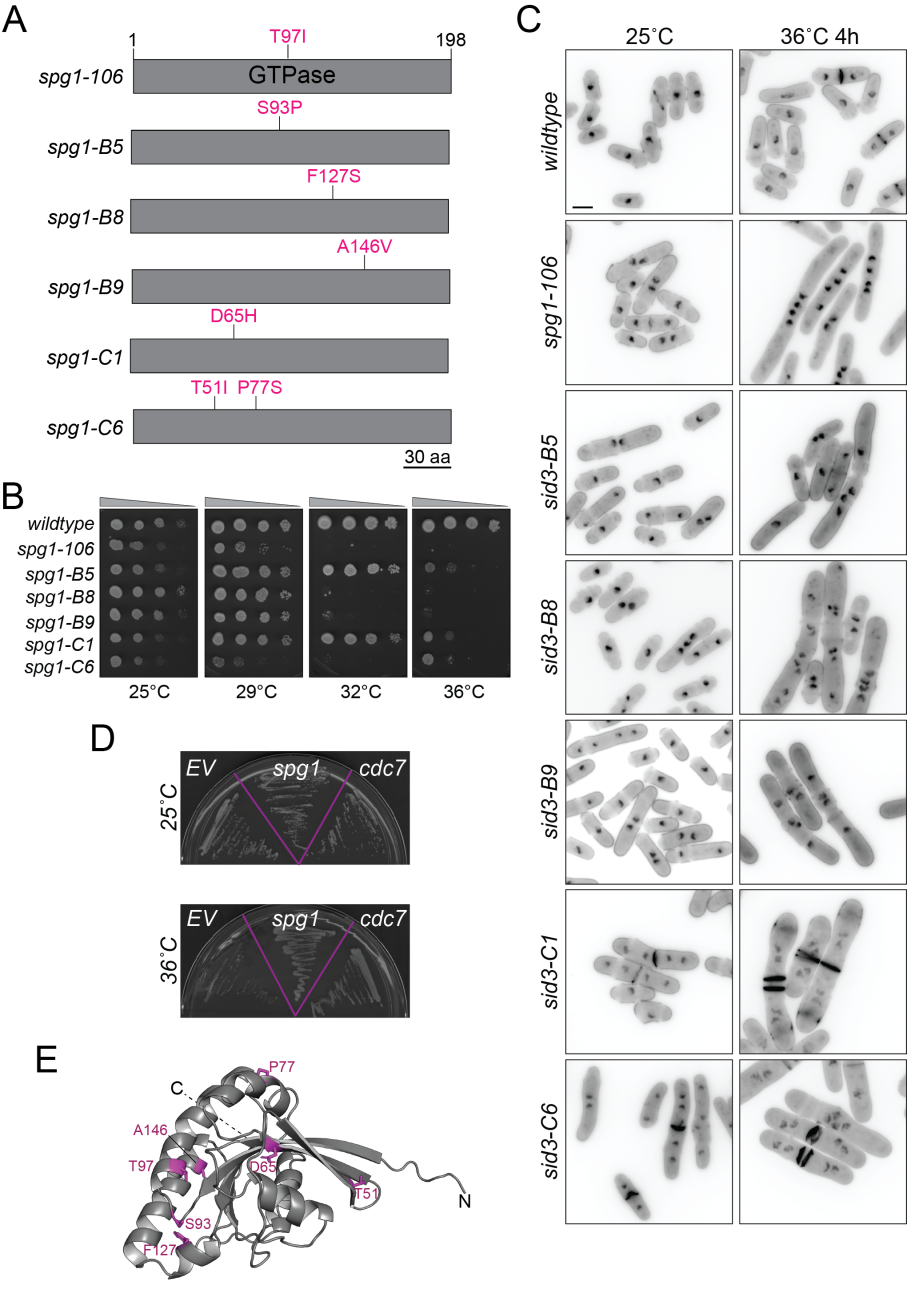
(A) Schematics of the GTPase Spg1, drawn to scale. The mutations encoded by the listed temperature sensitive alleles are labelled in magenta. (B) The indicated strains were all grown in liquid YE at 25°C until they reached mid-log phase. Then, 10-fold serial dilutions were made and 2.5 µL of each was spotted on YE agar plates and incubated at the indicated temperatures for 2-5 days prior to imaging. (C) The indicated strains were grown at 25°C and shifted to 36°C for 4 hours. Samples were collected at both temperatures and cells were fixed and stained with DAPI and methyl blue. Scale bar, 5 µm. (D) *spg1-C1 ura4-D18* cells were transformed with plasmids expressing empty vector (pUR19), pUR19- *spg1,* or pUR19- *cdc7* . Transformants were then streaked to minimal media plates lacking uracil and incubated at 25˚C or 36˚C for 4 days. (E) Ribbon diagram of a structural model of *S. pombe* Spg1 using AlphaFold2 (Jumper et al., 2021; Varadi et al., 2022) with positions of mutations indicated in magenta.

## Description

In the yeast *Schizosaccharomyces pombe* , cytokinesis is enabled by an actin- and myosin-based cytokinetic ring (CR) coupled to formation of a division septum (Cheffings et al., 2016; Glotzer, 2017; Mangione and Gould, 2019) . A signaling pathway named the septation initiation network (SIN) is essential for normal CR assembly and maintenance, constriction, and septation (reviewed in Cullati and Gould, 2019; Simanis, 2015; Xiao and Dong, 2021). At the top of the SIN cascade is the Spg1 GTPase (Schmidt et al., 1997; Sohrmann et al., 1998) . When Spg1 is inactivated, SIN signaling and cytokinesis fail, and this produces inviable cells that are elongated and multinucleated. Reciprocally, enhanced Spg1 activity induces the formation of multiple CRs and septa in the absence of nuclear division and is also lethal.

Here, we examine six previously uncharacterized temperature-sensitive strains obtained in a screen for mutants that fail cytokinesis and septation (Balasubramanian et al., 1998) . By genetic analysis, these strains were found to be in the same complementation group. Further, none of these mutants segregated in genetic crosses away from *spg1-106* and it was concluded that these six strains were additional mutant alleles of *spg1* (Balasubramanian et al., 1998) *.*

To further investigate the nature of these alleles, the *spg1* open reading frame was amplified from each of the six strains and sequenced to determine what mutations were present. In each case, one or more mutations were detected ( Figure 1A ). Two alleles ( *spg1-B5* and *spg1-B10* ) had the same mutation causing a serine to proline change at residue 93 (S93P). Two other alleles ( *spg1-B9* and *spg1-B11* ) contained an alanine to valine substitution at position 146 (A146V). A fifth allele ( *spg1-C1* ) had a change at residue 65 from aspartic acid to histidine (D65H). A sixth allele ( *spg1-C6* ) contained a threonine to isoleucine change at residue 51 and a proline to serine substitution at residue 77. Finally, we also sequenced the *spg1-106* and *spg1-B8* open reading frames because the mutations in these alleles had not previously been reported and found a threonine to isoleucine change at position 97 in *spg1-106* and a phenylalanine to serine change at position 127 in *spg1-B8* ( Figure 1A ). Thus, there are now six known and distinct temperature-sensitive mutant alleles of *spg1* .

We characterized the four new alleles further (eliminating the duplicative *spg1-B10* and *spg1-B11* alleles from further analysis) by first determining the range of temperature-sensitivity of each by spotting at a variety of temperatures. All temperature-sensitive alleles grew less than wildtype at 36°C. At 32°C, most alleles grew less than wildtype except for *spg1-B5* and *spg1-C1,* and *spg1-106* and *spg1-C6* also showed reduced growth at 29°C ( Figure 1B ). Interestingly, *spg1-C6* reproducibly grew better at 36˚C than 32˚C ( Figure 1B ). To visualize the cell phenotypes, we examined each mutant by staining for nuclei and septa after the cells were grown at 25°C and then shifted to 36˚C for 4 hours. We found that wildtype, *spg1-106* , *spg1-B8,* *spg1-B5* and *spg1-B9* looked similar to wildtype at 25°C, but at 36°C the cells were multinucleated with no septum present ( Figure 1C ), as has been previously reported for *spg1-106* (Balasubramanian et al., 1998) . Interestingly, *spg1-C1* and *spg1-C6* , even at 25°C, had many multinucleated cells with one medial septum. Additionally, at 36°C most cells were multinucleated with 1 or 2 medial septa ( Figure 1E ). We conclude that all *spg1* alleles have defects in cell division.

We next tested if each new mutant could be rescued by wildtype *spg1 ^+^* . Indeed, a *spg1 ^+^* genomic clone contained within pUR19 (Barbet et al., 1992) rescued the temperature-sensitive growth of all four mutants, illustrated by the rescue of *spg1-C1* in Figure 1D . The *spg1-106* allele can also be rescued by over-expression of Cdc7 , a protein kinase that it activates for SIN signaling (Balasubramanian et al., 1998) . We found that all four mutants were also rescued by over-expression of Cdc7 , illustrated by the rescue of *spg1-C1* in Figure 1D .

We also modeled the encoded mutations onto the AlphaFold2 predicted Spg1 protein structure (Jumper et al., 2021; Varadi et al., 2022) ( Figure 1F ). It will be interesting to investigate if the alleles with distinct phenotypes from *spg1-106* ( *spg1-C1* and *spg1-C6* ) are simply milder alleles or if perhaps they have different molecular defects. Deciphering if GTP binding, GTP hydrolysis, partner binding or a different function are disrupted in the proteins encoded by these alleles will be exciting to determine in future studies.

## Methods

Yeast methods

*S. pombe* strains were grown in yeast extract (YE) and standard *S. pombe* mating, sporulation, and tetrad dissection techniques were used to construct new strains (Moreno et al., 1991) .

Molecular biology methods

Plasmids were constructed using standard molecular biology techniques. *spg1* alleles were sequenced by generating a PCR product with an oligonucleotide 72 bp upstream of the start site (CGAACGAGGCTTCTTATCCT) and 47 bp downstream of the stop codon (ACCCACACTCCTTTATCATG) (Integrated DNA technologies). The PCR product was sequenced with an additional forward oligonucleotide at 33 bp upstream of the *spg1* start site (ATCTACTGCCGGGGTTCAAG).

Microscopy and image analysis

Strains for fixed-cell imaging experiments were grown at 25°C in YE and then shifted to 36°C for 3 hours. Cells were fixed with 70% ethanol for DAPI and methyl blue (MB) staining as described previously (Roberts-Galbraith et al., 2009) . Images were acquired using a Zeiss Axio Observer inverted epifluorescence microscope with Zeiss 63× oil (1.46 NA) objective and captured using Zeiss ZEN 3.0 (Blue edition) software. A singular medial Z slice was obtained. All images were further processed using ImageJ (Schindelin et al., 2012) .

## Reagents

The strains used in this study and their genotypes are listed below.

**Strain** **Genotype** **Source**

KGY1038 *spg1-B5 ura1 leu1-32 mam2::LEU2 ade6-M216 h90 (Balasubramanian et al., 1998)*

KGY1054 *spg1-B9 ura1 leu1-32 mam2::LEU2 ade6-M216 h90 (Balasubramanian et al., 1998)*

KGY1044 *spg1-C1 ura1 leu1-32 mam2::LEU2 ade6-M216 h90 (Balasubramanian et al., 1998)*

KGY1048 *spg1-C6 ura1 leu1-32 mam2::LEU2 ade6-M216 h90 (Balasubramanian et al., 1998)*

KGY246 *ade6-M210 leu1-32 ura4-D18* *h ^-^* Lab stock

KGY8016 *spg1-B8 ade6-M210 h ^-^* Lab stock

KGY1103 *spg1-106 ade6-M21X ura4-D18 leu1-32 h ^-^* Lab stock

KGY4491-2 *spg1-B5 ade6-M21X leu1-32 ura4D-18 h ^-^* This study

KGY4683-2 *spg1-B9 ade6-M21X leu1-32 ura4D-18 h ^-^* This study

KGY4879-2 *spg1-C1 ade6-M21X leu1-32 ura4D-18 h ^+^* This study

KGY5068-2 *spg1-C6 ade6-M21X leu1-32 ura4D-18 h ^-^* This study
